# An Address Delivered at the Fourth Anniversary Meeting of the Medical and Surgical Association, July 20, 1836

**Published:** 1837-01

**Authors:** 


Art. XIII.—*An Address delivered at the Fourth Anniversary Meeting of the Medical and Surgical Association, July* 20, 1836. By J. G. Crosse, Esq., Surgeon to the Norfolk and Norwich Hospital, &c. &c. —*Worcester*, 1836. 8vo.

Just as we were closing our Notices, the above publication reached us. We have only left ourselves space to say, that it is a very learned and excellent performance, and worthy of taking its place with the Addresses delivered before the Association on former years. We are gratified to notice the rapid and progressive extension of this most excellent Society.

